# The socio-economic inequality in body mass index: a PERSIAN cohort-based cross-sectional study on 20,000 Iranian adults

**DOI:** 10.1186/s12902-022-01096-2

**Published:** 2022-07-15

**Authors:** Farhad Pourfarzi, Satar Rezaei, Telma Zahirian Moghadam, Hamed Zandian, Foad Dibazar

**Affiliations:** 1 Digestive Disease Research Center, Ardabil University of Medical Sciences, Ardabil, Iran; 2 Research Center for Environmental Determinants of Health, Health Institute, Kermanshah University of Medical Sciences, Kermanshah, Iran; 3 Social Determinants of Health Research Center, Ardabil University of Medical Sciences, Ardabil, Iran; 4 Department of Community Medicine, School of Medicine, Ardabil University of Medical Sciences, Ardabil, Iran

**Keywords:** Body mass index (BMI), Socio-economic status (SES), Inequality, Obesity, Decomposing

## Abstract

**Introduction:**

The aim of the present study was to explore and determine the association between BMI and socio-economic factors in Iran.

**Methods:**

Adults aged 35 to 70 (*n* = 20,460) were included from Ardabil Non-Communicable Disease (ArNCD) cohort study. BMI was calculated as kg/m^2^. Principal Component Analysis (PCA) was used to determine the socio-economic quintiles. Multivariate linear regression was performed to analyze the association of BMI as dependent variable with explanatory variables, Additionally, decomposition analyzing conducted to identify factors that explained wealth-related inequality in obesity.

**Results:**

The prevalence of overweight and obese people was 83.7% (41.4% overweight and 42.5% obese) wherein the highest frequency of obese people belonged to the age group of 45 to 49 years old (19.9%) and to the illiterate people (33.1%). The results of multivariate linear regression model showed that age, being female, marriage, lower education level, having chronic disease, alcohol use, and higher socioeconomic level positively associated with obesity. The results of the decomposition model showed that the most important variables affecting socioeconomic inequality in higher BMI level were socioeconomic status (75.8%), being women (5.6%), education level (− 4.1%), and having chronic disease (2.4%).

**Conclusion:**

BMI showed significant association with socio-economic status, where richest people had significantly higher BMI than poorest group. Considering the direct role of high BMI in non-communicable diseases, new policies are needed to be developed and implemented by means of diet intervention and increased physical activity to control the BMI in the population of Iran.

## Introduction

Obesity is a major public health problem worldwide and its prevalence varies in different parts of the world and even in different groups [1]. Obesity was once common in high-income countries, however it is currently common in low-income countries [2]. The World Health Organization (WHO) estimates that globally obesity affects 500 million people and could be increased to one billion by 2030 [3].

Obesity is known as one of the main risk factors of type 2 diabetes, asthma, hypertension, stroke, coronary artery disease, cancer and related mortality, liver and gallbladder disease, sleep apnea, osteoarthritis, and gynecological diseases [4]. The health-care costs are also of concern. For instance, in the United States, total obesity-related expenditures account for 1.9% of GDP [5].

Obesity is generally caused by imbalanced intake and consumed calories. However, the cause of obesity is still unclear and is associated with a series of factors including genetics, health-related behaviors such as diet and physical activity, psychological, social, and economic factors [6, 7].

Despite widespread individual-level interventions, the obesity epidemic remains uncontrolled. Following this consensus, community-based approaches, which are opposed to the individual, can add to traditional individual interventions. In fact, large number of socio-demographic and socioeconomic indicators are associated with obesity [8].

Socio-economic inequalities in health are chiefly resulted from the socio-economic factors such as income, education, and employment status on health condition and also on account of the mediating factors such as destructive health behaviors and poor living conditions [9]. However, it should be pointed out that the nature of the relationship between obesity and socio-economic factors differs in developed and developing countries [10].

In developed countries, as per various studies, obesity widely affects people with lower SES while families with higher SES follow healthy diets. However, low-income families often choose more energetic foods to provide higher energy at lower cost. On this basis, the causes of obesity should be considered beyond individual factors in order for interventions for successful prevention and control of obesity [11].

Since recognizing the factors affecting obesity in the communities can lead to better formulation of obesity prevention and control policies, the assessment of socio-economic causes of obesity in Iran is of great importance.

## Methods

### Study setting and sample

This population-based cross-sectional study was conducted in Ardabil (the capital of Ardabil province) in the northwest of Iran. Ardabil has a population of approximately 610,000 people [12]. This study uses data extracted from the Prospective Epidemiological Research Studies in IrAN (PERSIAN) [13], which was conducted to develop the context needed to modify health-care policies in the field of Non-communicable diseases (NCDs). The PERSIAN cohort is a cohort sample of various sites across Iran. Ardabil Non-Communicable Disease (ArNCD) cohort study is one of the 18 geographically distinct study areas of PERSIAN cohort study.

The study participants included 20,525 adults between the ages of 35–70 years from both men and women, mainly Azari ethnicity, and whom living in the city of Ardabil, north-west of Iran. Based on PERSIAN cohort protocol, enrolled participants have to pass several steps including clinical test, anthropometric evaluation, medical, nutritional, and mental evaluation, etc. People with disability such as deaf, blind, palsy and people with mental disorders, mental retardation, and any psychiatric illness in the acute phase; were excluded from the study. The details of the sampling design can be found elsewhere [13]. Accounting for missing data, the final sample size of the study was 20,460 people.

### Data and variables

Data gathering was conducted from May 2017 to February 2020. Trained interviewers administered the questionnaire. Obesity as the dependent variable of our study was assessed based on BMI (continuous). Weight and height of the participants were measured based on American National Institute of Health (NIH). To measure weight and height, 111 Saka standing hand scales and 431 Saka wall height gauges were used, respectively. To report obesity status, BMI was classified based on the American College of Cardiology and the American Heart Association category into four groups; BMI less than 18.5 labeled as underweight, BMI between 18.5 to < 25 as the healthy weight range (normal), BMI between 25.0 to < 30 was the overweight range, and BMI from 30.0 and higher was ranged as obese. This category is defined regardless of age range [14]. Age (categorized from under 40 to upper 65), sex (male/female), marital status (single/ married/ divorced/ Other), education status (illiterate/ primary/ tips/ diploma/ academic degree) and occupation status were the independent variable in this study. Data on the non-communicable diseases (cardiovascular disease, diabetes, hypertension, and other disease including stork, renal failure, Hepatitis (B /C), Epilepsy, Amnesia, MS, etc.) was extracted based on the individuals’ self-declaration, clinical tests results, and request to see their clinical records. In addition, the wealth index, as the socio-economic status of the participants, was calculated based on their self-reported wealth, and it was divided into five quintiles from 1st quintile as the poorest to 5th quintile as the richest groups.

This study used the Principal Component Analysis (PCA) technique [15] to estimate the socioeconomic status of the study participants. Filmer and Pritchett (2001) popularized the use of PCA for estimating wealth levels using asset indicators to replace income or consumption data [16]. The estimation of relative wealth using PCA is based on the first principal component. Formally, the wealth index for household i is the linear combination,

$${y}_i={a}_1\left(\frac{x_1-{\overline{x}}_1}{s_1}\right)+{a}_2\left(\frac{x_2-{\overline{x}}_2}{s_2}\right)+\dots +{a}_k\left(\frac{x_k-{\overline{x}}_k}{s_k}\right)$$

Where, ${\overline{x}}_k$ and *s*_*k*_ are the mean and standard deviation of asset *x*_*k*_, and α represents the weight for each variable *x*_*k*_ for the first principal component. By definition the first principal component variable across households or individuals has a mean of zero and a variance of λ, which corresponds to the largest eigenvalue of the correlation matrix of *x*. The first principal component or wealth index can take positive as well as negative values. Assets and housing characteristics (e.g. housing situation, number of bedrooms at home, family assets, etc.), education level, and job were the explanatory variables in the PCA. Based on the wealth score, samples were divided into five quintiles from the poorest to the richest (1st quintile as the poorest and 5th quintile as the richest) as socioeconomic status.

### Statistical analysis

Statistical analyses were performed using the Stata 17 for Windows running on 64-bit versions of Windows 10 (College Station, Texas, USA). Descriptive statistics for obesity indices were calculated for both men and women. Differences in continuous variables between genders were tested by Student’s *t* test. Differences in binomial categorized variables between men and women were analyzed by Pearson’s *χ* 2 test including marital status (single/married/divorced/other), educated (illiterate/primary/tips/diploma/academic), diabetes (yes/no), hypertension (yes/no), cardiac ischemic (yes/no), and socioeconomic quintile (from poorest to richest in five quintiles).

Multivariate linear regression was performed to analyze the association of BMI as dependent variable with explanatory variables. Again, anthropometric indices for obesity were not tested together in the same regression model due to the high multicollinearity. All *P* values reported are two-tailed and *P* < 0·05 was considered as statistically significant.

The multiple linear regression model Matrix form writes for all n points simultaneously:

$$y= Xb+e$$

$$\left(\begin{array}{c}{y}_1\\ {}{y}_2\\ {}{y}_3\\ {}\dots \\ {}{y}_n\end{array}\right)=\left(\begin{array}{c}1\kern1em {X}_{11}\kern0.75em {X}_{12}\kern0.5em \dots \kern0.75em {X}_{1p}\\ {}1\kern1em {X}_{21}\kern0.75em {X}_{22}\kern0.5em \dots \kern0.75em {X}_{2p}\\ {}1\kern1em {X}_{31}\kern0.75em {X}_{32}\kern0.5em \dots \kern0.75em {X}_{3p}\\ {}\dots \\ {}1\kern1em {X}_{n1}\kern0.75em {X}_{n2}\kern0.5em \dots \kern0.75em {X}_{np}\end{array}\right)\left(\begin{array}{c}{b}_0\\ {}{b}_1\\ {}{b}_2\\ {}\dots \\ {}{b}_p\end{array}\right)+\left(\begin{array}{c}{e}_1\\ {}{e}_2\\ {}{e}_3\\ {}\dots \\ {}{e}_n\end{array}\right)$$

where

y = (y_1_;::; y_n_) ∈*ℝ*^*n*^ is the *n* × 1 response vector

X = [1_n_; x_1_;:; x_p_] ∈*ℝ*^*n* × (*p* + 1)^ is the *n* × (*p* + 1) design matrix

_ 1_n_ is an *n* × 1 vector of ones

_ x_j_ = (x_1j_;:::; xnj) ∈*ℝ*^*n*^ is j-th predictor vector *n* × 1

b = (b_0_; b_1_;:::; b_p_) ∈*ℝ*^*n* × (*p* + 1)^ is (*p* + 1) × 1 vector of coefficients

e = (e_1_;:::; e_n_) ∈*ℝ*^*n*^ is the *n* × 1 error vector [17].

#### Measuring and decomposing socioeconomic inequality in alcohol use

Multivariate logistic regression was used to describe the relationships between BMI and explanatory variables. The study examined the socioeconomic differences in BMI among participants using the Relative Concentration Index (RCI) and Concentration Curve (CC) [18]. We used the relative concentration index (RCI) to measure and decompose the socioeconomic inequality in BMI among Ardabil adults (35 to 70 years of age). In addition, we used Concentration Curve (CC) to investigate the socioeconomic inequality in BMI graphically. The CC plots the cumulative percentage of socioeconomic status ranked participants on the x-axis and the cumulative percentage of the health outcome (BMI in our case) on the y-axis. The curves deviation from the line of equality indicates the severity of inequality. The RCI is equivalent to twice the area between the perfect equality line (45-degree line) and the concentration curve [19]. RCI values range from − 1 to + 1. RCI is positive (negative) when the concentration curve lies below (above) the line of perfect equality. The RCI’s positive (negative) value indicated that the BMI value concentrated more among the richest (poorest) [18]. Following Wagstaff [20], RCI was separated by $\frac{1}{1-\mu }$ for normalization. For this calculation, μ is assumed the measure of BMI. Eventually, the process of decomposition was used to classify the key determinants of the reported inequities of BMI [21].

1 $$y=\alpha+{\textstyle\sum_k}\beta_kx_k+\varepsilon$$

where *x*_*k*_ describes the explanatory variables discussed in the previous section. Thus the RCI for BMI has been decomposed as follows [22]:

2 $$RC={\textstyle\sum_k}\left(\frac{\beta_k{\overline x}_k}\mu\right){RC}_k+\frac{AC_\varepsilon}\mu$$

Where *RC* is the relative concentration index for BMI, ${\overline{x}}_k$ the mean of *x*_*k*_ determinants, *C*_*k*_ are the *RC* for explanatory variables, and ${x}_k\left(\frac{\beta_k{\overline{x}}_k}{\mu}\right){RC}_k$ is the elasticity of BMI in relation to the explanatory variable *x*_*k*_ . $\sum_k\left(\frac{\beta_k{\overline{x}}_k}{\mu}\right){RC}_k$ presents the contribution of the explanatory factor *x*_*k*_ to the *RC*. The last term, $\frac{AC_{\varepsilon }}{\mu }$, is the residual component. Since RCI normalised our calculation of inequality, we used the following theorem in the decomposition analysis [22].

$$RC=\frac{\sum_k\left(\frac{\beta_k{\overline{x}}_k}{\mu}\right){RC}_k}{1-\mu }+\frac{\frac{AC_{\varepsilon }}{\mu }}{1-\mu }$$

## Results

Out of 20,480 people in the study, 15,570 (76.1%) were overweight (25 < BMI ≤ 30) and obese (BMI > 30), where about 44.5% of them was obese and 31.6% of them was over weighted people. The highest frequency of obese people belonged to the age group of 45 to 49 years (47.92%). According to the results, women were more obese than men were and the prevalence of obesity was higher among married people than other groups. Moreover, the prevalence of obesity among illiterate people was higher than other educational groups (35.3%). In the case of the underlying diseases, the prevalence of obesity among people with diabetes, hypertension, and cardiovascular disease was 45.25, 51.94, and 47.21%, respectively (Table 1).

**Table 1:** Socio-demographic characteristic of the study participants (*n* = 20,460)

	Obese and overweight (*n* = 15,570)		Non-obese (*n* = 4890)		Total	
	n	%	N	%	n	%
Age Categories						
< 40	2398	7459	817	25.41	3215	15.71
40–44	3013	75.36	985	24.64	3998	19.54
45–49	3158	7821	880	2179	4038	19.74
50–54	2814	79.16	741	20.84	3555	17.38
55–59	2039	75.02	679	24.98	2718	13.28
60–64	1407	74.88	472	25.12	1879	9.18
> 65	741	7010	316	29.90	1057	5.17
Gender						
Female	8768	79.11	2315	20.89	11,083	54.17
Male	6802	72.54	2575	27.46	9377	45.83
Marital Status						
Single	194	56.89	147	43.11	341	1.66
Married	14,228	76.45	4383	23.55	18,611	90.9
Other	1148	76.13	360	23.87	1508	7.37
Education						
illiterate	4536	69.46	1994	30.54	6530	31.92
Primary	3279	71.21	1326	28.79	4605	22.51
Tips	2371	77.46	690	22.54	3061	14.96
Diploma	2639	76.27	821	23.73	3460	16.91
Academic	2145	76.50	659	23.50	2804	13.70
Chronic Disease						
Have Diabetes	1858	77.84	529	22.16	2387	11.67
Have Hypertension	3490	82.18	757	17.82	4247	2076
Have Cardiac Ischemic	1358	78.09	381	21.91	1739	8.50
Smoking						
No	13,373	77.89	3796	22.11	17,169	83.91
Yes	2197	66.76	1094	33.24	3291	16.09
Alcohol						
No	14,730	76.02	4646	23.98	19,376	94.70
Yes	840	77.49	244	22.51	1084	5.30
Socioeconomic Quintiles						
Poorest	2428	59.34	1664	40.66	4092	20.0
Poor	1937	47.34	2155	52.66	4092	20.0
Middle	3459	84.53	633	15.47	4092	20.0
Rich	3118	76.02	984	23.98	4102	20.05
Richest	3428	83.98	654	16.02	4082	19.95

**Fig. 1:**
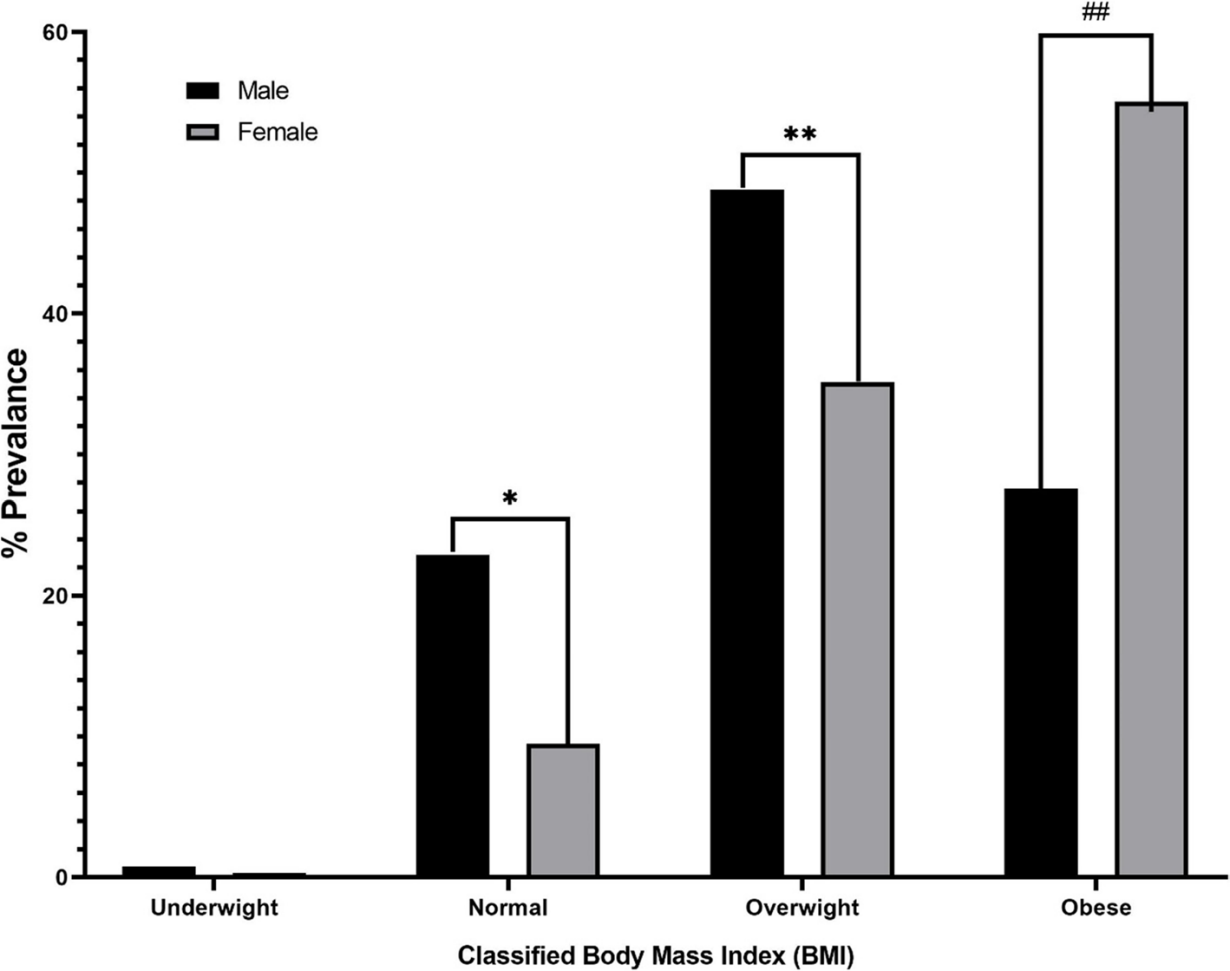
Dispersion of men and women participating in the study by Body Mass Index (BMI) classes. There is a significant difference between women and men in terms of BMI, where the distribution of men is significantly higher than women is in the category of * = normal and ** = overweight groups and in contrast to the distribution of women is more than men is in the group of ## = obese people

Figure 1 showed that there was significant difference between men and women in term of BMI classes, where the prevalence of overweight in men is higher than women and the prevalence of obesity in women is higher than man (*p* < 0.001).

The results of multivariate linear regression in Table 2 showed the relationship between obesity (higher level of BMI) and demographic/socio-economic factors. The multivariate linear regression model showed that the coefficient of determination (R Square) was equal to 0.397 and the coefficient of modification (Adjusted R Square) was equal to 0.396. The proximity of these two values indicated that the variables used in the model were able to work well and provided a good fit. Although, age was significantly associated with obesity, but its association decreased by increasing age and was not significant in group aged over 65 years (*p* = 0.836). Female gender was significantly associated with BMI (2.546, 95% CI: 2.691 to 2.40, *p* < 0.001). In addition, a significant and positive relationship was predicted between marital status and BMI in which married people were more obese than single group. According to the results, BMI was decreased in individuals with increased education level and there was a significant and inverse (negative) relationship between education and BMI (− 1.425, 95% CI: − 1.679 to − 1.170, *p* < 0.001). Furthermore, as per results, diabetes and hypertension were significantly associated with increased BMI so that people with diabetes and CVDs were less likely to be obese and overweight than people without chronic disease were. People with high blood pressure were likely to increase BMI and be overweight and obese. Smoking had significant correlation with lower BMI, in contrast, alcohol use positively correlated with high BMI. Additionally, there was a significant and positive relationship between socio-economic status and BMI wherein rich people had significantly higher BMI (6.204, 95% CI: 5.971 to 6.438, *p* < 0.001).

**Table 2:** Effects of socioeconomic and demographic factors on BMI using multilevel linear regression

	Coefficients	[95% CI]	SE	t	*P*-value
Age					
< 35 (ref.)					
40–44	0.467	0.264 to 0.670	0.103	4.52	< 0.001
45–49	0.885	0.677 to 1.094	0.106	8.33	< 0.001
50–54	0.932	0.712 to 1.151	0.112	8.31	< 0.001
55–59	0.629	0.388 to 0.870	0.122	5.12	< 0.001
60–64	0.363	0.091 to 0.636	0.139	2.62	0.009
> 65	−0.034	−0.367 to 0.297	0.169	−0.21	0.836
Sex					
female	2.546	2.691 to 2.40	0.074	34.31	< 0.001
Marital Status					
Single (ref.)	–	–	–	–	–
Married	2.429	1.960 to 2.897	0.239	10.16	< 0.001
Other	2.530	2.012 to 3.049	0.264	9.57	< 0.001
Education					
Illiterate (ref.)	–	–	–	–	–
Primary	−0.324	−0.500 to − 0.148	0.089	−3.61	< 0.001
Tips	−0.425	− 0.637 to − 0.214	0.107	−3.95	< 0.001
Diploma	−1.09	−1.307 to − 0.872	0.110	−9.84	< 0.001
Academic Degree	−1.425	−1.679 to −1.170	0.129	−10.97	< 0.001
Chronic Disease					
Have Diabetes	0.334	0.141 to 0.526	0.098	3.40	< 0.001
Have Hypertension	1.505	1.343 to 1.667	0.082	18.21	< 0.001
Have Cardiac Ischemic	0.227	0.004 to 0.450	0.113	2.00	0.045
Smoking					
No (ref.)	–	–	–	–	–
Yes	−0.48	−0.664 to −0.295	0.094	−5.09	< 0.001
Alcohol					
No (ref.)	–	–	–	–	–
Yes	0.523	0.242 to 0.803	0.143	3.65	< 0.001
Socioeconomic Quintiles					
Poorest (ref.)	–	–	–	–	–
Poor	−1.028	−1.217 to −0.839	0.096	−10.67	< 0.001
Middle	3.538	3.344 to 3.732	0.099	35.67	< 0.001
Rich	5.266	5.061 to 5.470	0.104	50.52	< 0.001
Richest	6.204	5.971 to 6.438	0.119	77.21	< 0.001

*CI* Confidence interval, *SE* Standard Error, *BMI* Body Mass Index, *ref* Reference group

**Fig. 2:**
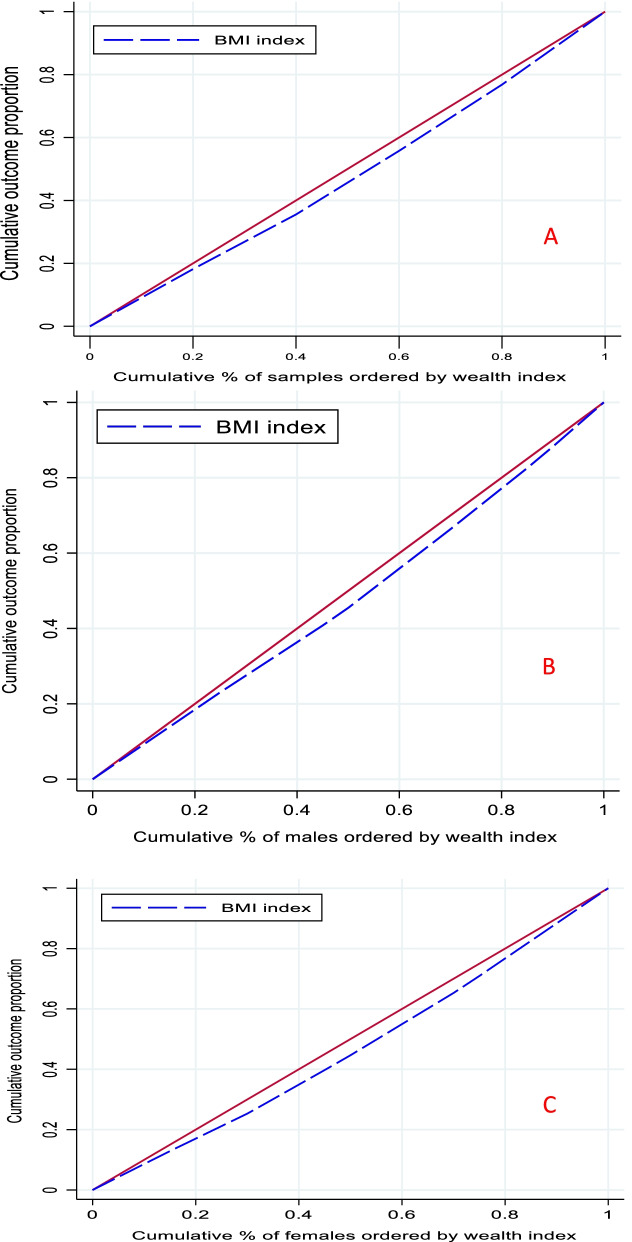
Concentration curve for BMI among total samples (**A**), males (**B**) and females (**C**) based on the wealth index

The results related to socioeconomic inequality in BMI by gender in the study population are reported in Table 3 and Fig. 2. The estimated Cn was 0.054 (95% confidence interval [CI]:0.053–0.055) for the entire population, 0.050 (95% CI: 0.049–0.052) for men and 0.068 (95% CI: 0.066 to 0.070) for women. This estimation shows that higher BMI is more common among people with higher socioeconomic status. Socioeconomic inequality in obesity was significant for both men and women, but the severity of inequality among women was higher than men was. The concentration curves are also shown in Fig. 2. According to Fig. 2, BMI concentration curves for the study population, men and women are below the equality line, indicating that higher BMI is more common among wealthy people.

**Table 3:** Normalized concentration index for BMI for male, female and whole of sample

	Relative concentration index	Confidence interval 95%	*p*-value
Female	0.0685	0.0669 to 0.0700	< 0.0001
Male	0.0509	0.0492 to 0.0527	< 0.0001
Whole of sample	0.0547	0.0534 to 0.0559	< 0.0001

Table 4 shows the decomposition analysis results of inequality in BMI in the study population. The findings related to the final effects showed that old age and higher education level (academic degree) have negative relationship with obesity (higher BMI). There is also a direct (positive) relationship between higher BMI with other variables such as female gender, marital status, alcohol use, having chronic disease, and higher economic status. The results of the decomposition model showed that the most important variables affecting socioeconomic inequality in higher BMI level were socioeconomic status (75.8%), being women (5.6%), education level (− 4.1%), and having chronic disease (2.4%). The results suggested that 82.1% of socioeconomic-related inequality in higher BMI level were explained by variables included in the study and the remaining 17.9% was associated with the variables that were not included in our decomposition model.

**Table 4:** Decomposition to determine factors lie behind socioeconomic inequality in ArNCD cohort study

Variable	Concentration index	Elasticity	% Contribution	Summed%
Age (< 35 ref.)				
40–44	−0.0163	0.0025	−0.0734	0.8
45–49	0.0446	0.0059	0.4837	
50–54	0.0445	0.0055	0.4487	
55–59	−0.0040	0.0028	−0.0209	
60–64	−0.0310	0.0011	−0.0644	
> 65	−0.1160	−0.0001	0.0128	
Sex (male ref.)				
female	−0.0657	−0.0469	5.6411	5.6
Marital Status (Single ref.)				
Married	0.0083	0.0752	1.1471	0.9
divorced	−0.0325	0.0050	−0.2963	
Education (Illiterate ref.)				
Primary	−0.0926	−0.0025	0.4196	−4.1
Tips	0.0237	−0.0022	−0.0938	
Diploma	0.1061	−0.0063	−1.2148	
Academic Degree	0.2652	−0.0066	−3.2191	
Chronic Disease (have not ref.)				
Have Diabetes	0.0237	0.0013	0.0577	2.4
Have Hypertension	0.1163	0.0106	2.2635	
Have Cardiac Ischemic	0.0398	0.0007	0.0478	
Smoking (No ref.)				
Yes	−0.1310	−0.0026	0.6258	0.6
Alcohol (No ref.)				
Yes	0.0601	0.0009	0.1036	0.1
Wealth index (Poorest ref.)				
Poor	−0.4263	−0.0070	5.4501	75.8
middle	0.0397	0.0241	1.7468	
Rich	0.1753	0.0358	11.4820	
Richest	0.5020	0.0623	57.1676	
Explained	**82.1**			
Residuals	**17.9**			
Total	**100**			

## Discussion

The present study aimed to quantify and decompose socioeconomic inequalities in adult obesity in north-west of Iran. Using 20,460 PERSIAN cohort data from Ardabil, Iran, we analyzed being overweight and obese in Iranian adults aged 35 to 70 years. Socio-economic status was measured in overweight and obese people from Ardabil. Our descriptive results showed that 76.1% were overweight and obese in the population. However, in Ardabil, 32.6% were overweight and 15.9% were obese in 2000 [23]. Najdafi et al. showed that the overall prevalence of obesity and overweight in Iranian adults (aged 35 years and older) were 40.76 and 30.43%, respectively [24]. The increase in the prevalence of obesity indicated some changes in lifestyle and socio-economic factors in the region. Similarly, the prevalence of obesity in the United States, a country with a high prevalence of obesity and overweight, more than a third of adults were obese in 2010 [25]. In the adult population in Spain, the prevalence of obesity was 22.9 [26]. The prevalence of obesity in Turkey was 33.2% in women and 18.2% in men [27].

The comparison of the findings of the present study with the similar studies shows that the prevalence of obesity and being overweight was higher compared to other countries. Since obesity results from complex interactions among genetic, behavioral, cultural, and environmental factors, the impact of all these factors should be considered for the prevalence of obesity and being overweight in Iran [28]. The highest rate of obesity in this study could be attribute to the study population, where we conducted study on adults aged 35 and above years old. Inoue et al. showed that the prevalence of obesity is higher among adults than young [29].

Similar to previous studies in Iran [24, 30], the results of the present study showed that women were more obese than men. In previous studies, a number of factors such as unemployment, depression, unhealthy eating patterns, sleep disorders and illiteracy, low SES, number of pregnancies and physical inactivity have been identified as risk factors of obesity in women [31]. Generally, Iranian women have less physical activity than men due to limited social conditions and the type of outdoor clothing or the limited number of suitable gyms and sport clubs. In addition, childbirth can also be another reason. Various studies showed that women with lower levels of education, lower employment status, and lower incomes were more likely to be obese.

This study showed that higher education contribute with lower level of BMI. Alaba et al. showed that educational attainment was a major contributor to obesity in South Africa [32]. Similarly, Hajizadeh et al. explained income and education level as demographic variables as main factors of income-related inequality in obesity in Canada [33]. Additionally, education defined as a key contributors to inequalities in obesity in Spain [34]. In Iran, studies showed that people with lower levels of education have been more obese than their counterparts with higher levels of education [35]. As found in this study, the prevalence of obesity was higher among illiterate people than in other educational groups. Qualifications, as a form of cultural capital, may have consequences for the extent to which social standards of attractiveness and health messages about diet and physical activity are adhered to, thus emphasizing weight-loss [36, 37].

Moreover, marital status had a positive contribution to obesity wherein married adults were more obese than single people did. This finding was consistent with the results of other studies in Iran [35, 38–40]. Various studies have suggested some changes in the lifestyle and post-marital nutrition patterns as an influencing factor in increased BMI in adults. Findings of Azadbakht et al. (2005) showed that the percentage of energy and fat intake was higher among married people compared to single people [41]. Also, Sartorius et al. (2015) reported that single people spend more time exercising than married people [42]. Being married was detected as an important counteracting factor for high BMI in Sweden [36].

This study showed that people with diabetes were less likely to be obese and overweight. As shown in various studies, people with diabetes suffer from impaired insulin sensitivity to transport glucose to the cells of organs. Consequently, glucose remains in blood. When glucose, the fuel needed by the body, does not reach the cells, it causes weight loss, and as a result, diabetes causes a person to lose weight. Moreover, taking diabetic medications can lead to weight-loss in diabetics [43].

In the present study, people with high blood pressure were more likely to be overweight and obese. As per similar literature, high blood pressure in obese people was 2 to 6 times higher than people who were not overweight [44, 45].

Several studies that conducted a comprehensive assessment, showed a complex and controversial picture of socioeconomic inequalities in obesity in developing and developed countries. There are various studies in developed countries showed that the prevalence of obesity was common among people with low socio-economic status [46]. In contrast, there are evidences showed that the highest socioeconomic groups had the lowest prevalence of obesity [47]. According to the decomposition analysis of obesity inequality in Sweden, income was the main driving force behind obesity inequality [36]. In developed countries, socioeconomic inequalities in adult obesity is pro-rich. This is because low-income people in developed countries often use unhealthy foods, which are high in fat, sugar, and are cheaper for abdominal satiety. However, in developing countries, food consumption and obesity, which is a symbol of well-being in society, increase the prevalence of obesity in people with high socio-economic status [47, 48]. From the evidence, in several countries (including Europe, the United States, Australia, and Canada) the diet is changing economically and socially where people in higher social and economic groups tend to diet. Staying on a healthier diet using more fruits, vegetables, and less fat is common in rich groups. This indicated a person’s income or economic capability to buy healthy foods, which are normally more expensive than low-value food items [33, 49].

On the other hand, the present study in Iran as a developing country showed that rich people had significantly higher BMI. In Ardabil, obesity was statistically pro-poor, where it was in line with the previous studies in Iran. In agreement with our findings, previous studies in Iran have shown that obesity is less common in people with low SES and it is pro-poor. For example, Najafi et al. reported a lower prevalence of obesity among poor people in a sample of Iranians [50]. Mohammadi et al. (2011) found that income was positively associated with obesity [51]. Some Overall, our findings showed that higher SES was disproportionately responsible for being overweight and obese. Accordingly, crosscutting measures appear to be taken to control and prevent overweight and obesity among higher socioeconomic groups. Eliminating social and economic inequalities in health outcomes is the key public policy priority. Powerful support of government and targeted programs are needed to combat the obesity epidemic, reduce inequality in physical activity, and prepare the community for the associated consequences in the future. Eating behaviors and lifestyles in childhood are often followed in their future life. Hence, effective intervention programs to promote healthy lifestyles not only help fighting the obesity epidemic, but also prevent other chronic diseases, reduce future health-care costs, and pave the way for a healthier nation. 

The present study have had several advantages: the use of valid PERSIAN group data, which was obtained through determined and controlled methods and had a large sample size. In addition, individuals’ BMIs were obtained based on height and weight measurements at the Gastroenterology Research Center. Iranian society is not homogeneous and this can be clearly seen in the results of the study. It should be noted that the data on children and the population aged under 35 years old were excluded because children and age groups under 35 years were not included in the PERSIAN cohort sample population.

## Conclusion

The results showed an extremely high prevalence of obesity and being overweight in the studied area in comparison to other developing and also developed countries. BMI as the index of obesity was positively correlated with gender, marital status, education level, having chronic disease (hypertension, diabetes, and CVDs), smoking, alcohol use, and socioeconomic status. After creating socio-economic level for the population, BMI had a greater association with socio-economic status where the richest people had significantly higher BMI and socioeconomic inequality in obesity was pro-poor in this study. Considering the direct role of high BMI in non-communicable diseases and high mortality rate and also the direct and significant role of high socio-economic level in increased BMI and obesity, specific policies are needed to be developed and implemented through diet intervention and increased physical activity to control the increase in BMI of rich people. In addition, organized supports from health system with other social and economic sectors could be an effective policy strategy for reducing socioeconomic inequalities in obesity in adults.

## Data Availability

The datasets generated and/or analyzed during the current study are not publicly available due to PERSIAN cohort protocol (available at: https://persiancohort.com/) but are available from the corresponding author on reasonable request.

## Abbreviations

ArNCD: Ardabil Non-communicable Disease
BMI: Body Mass Index
SES: Socio-Economic Status
GDP: Gross Domestic Product
NCD: Non-communicable diseases
PERSIAN: Prospective Epidemiological Research Studies in IrAN
PCA: Principal Component Analysis
RCI: Relative Concentration Index
WHO: World Health Organization
